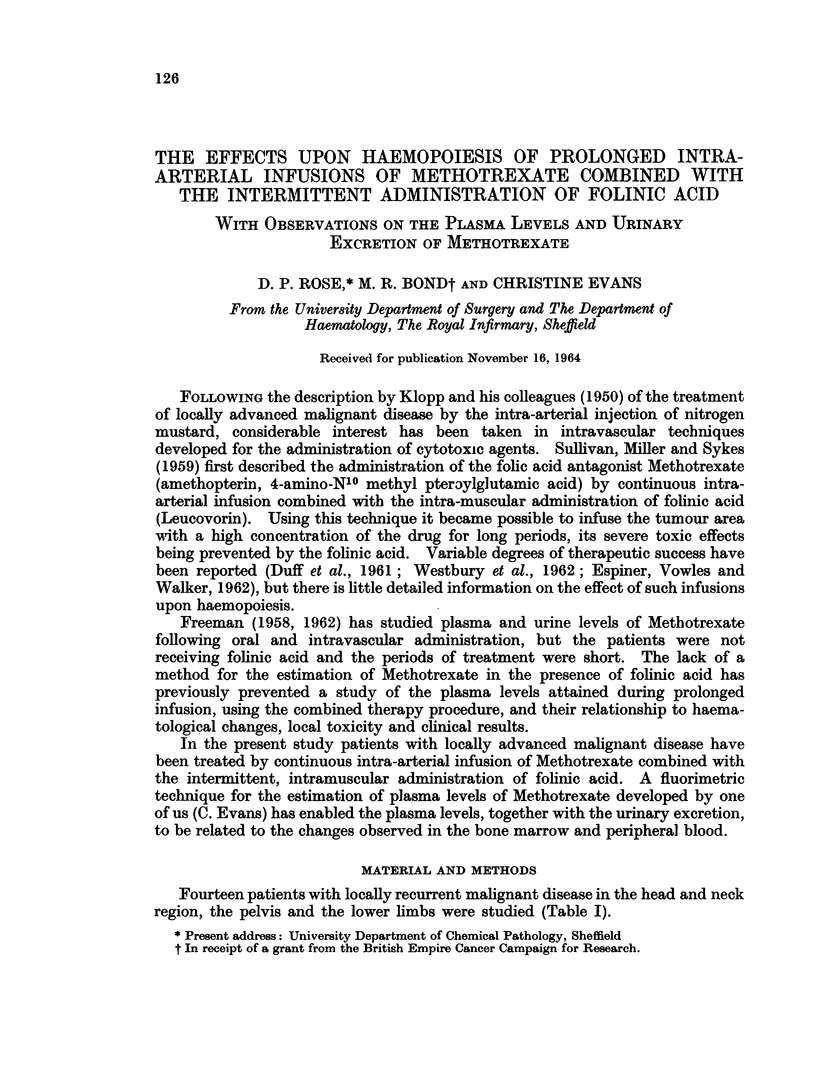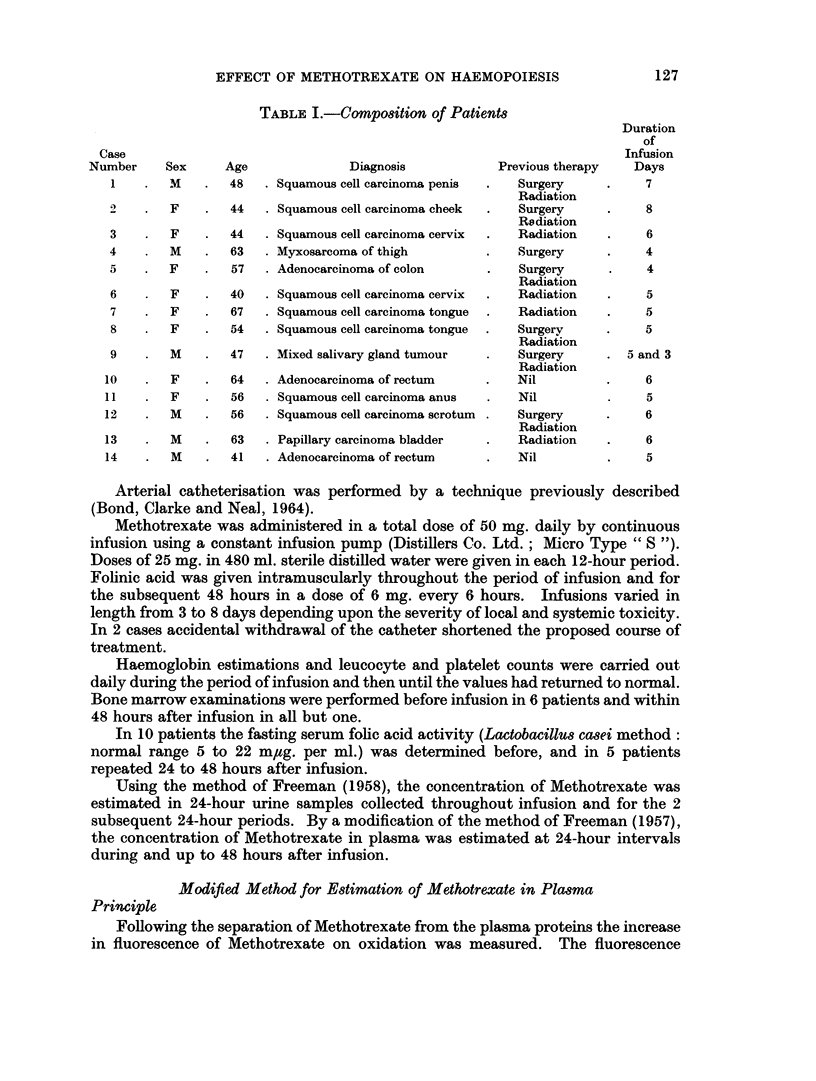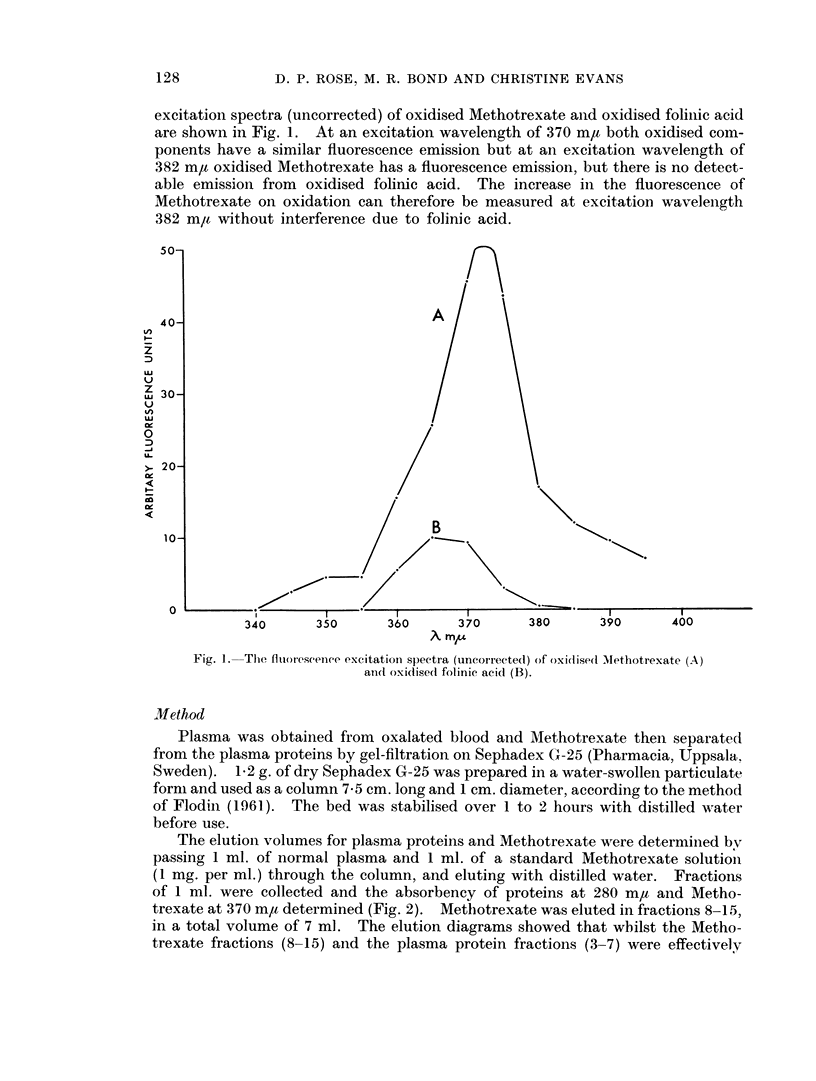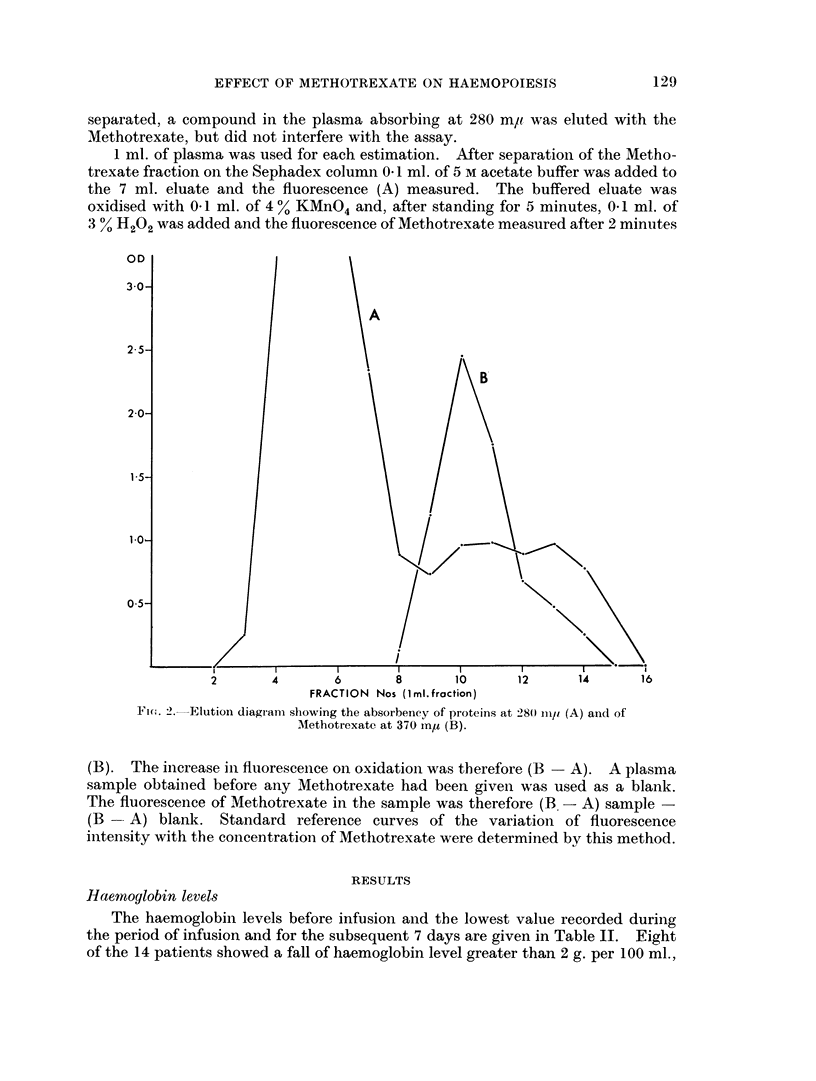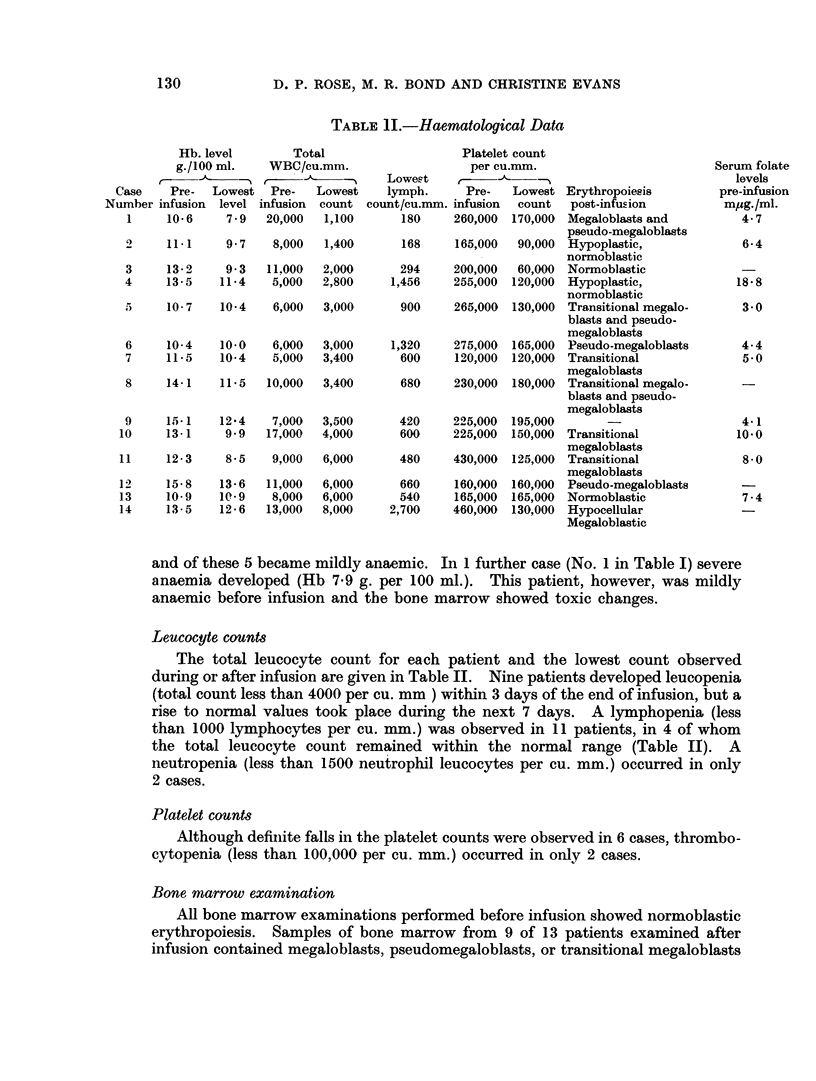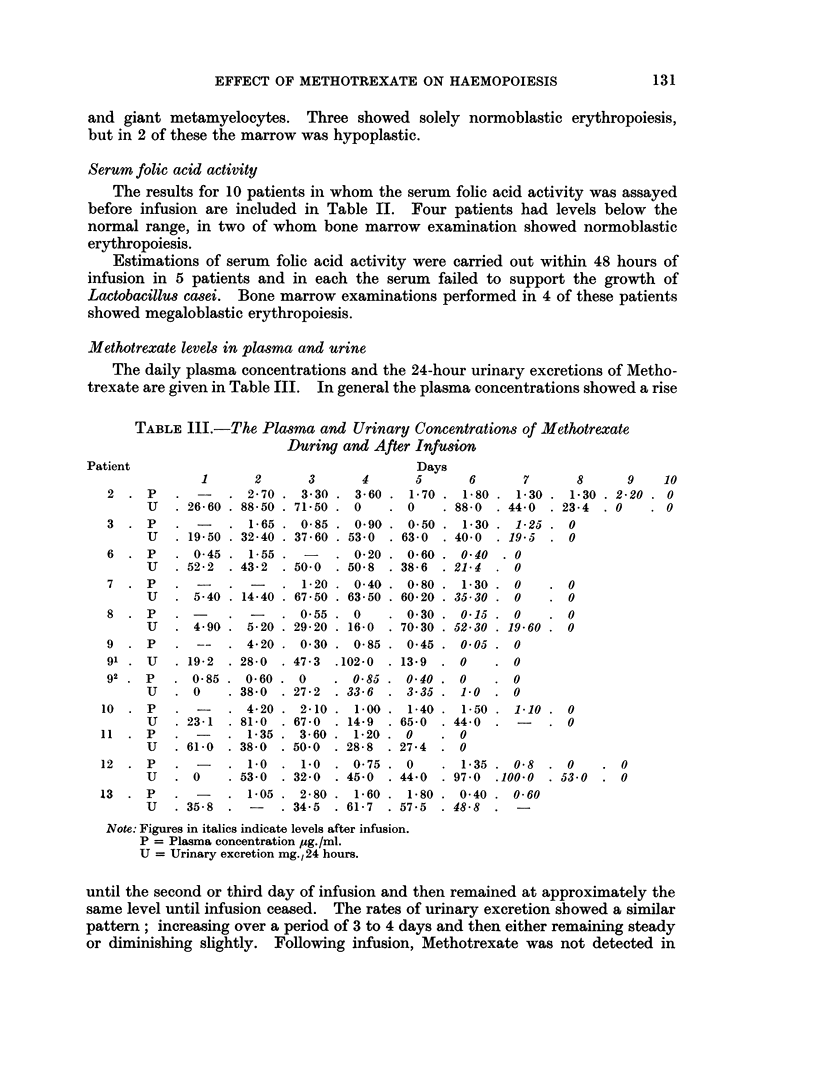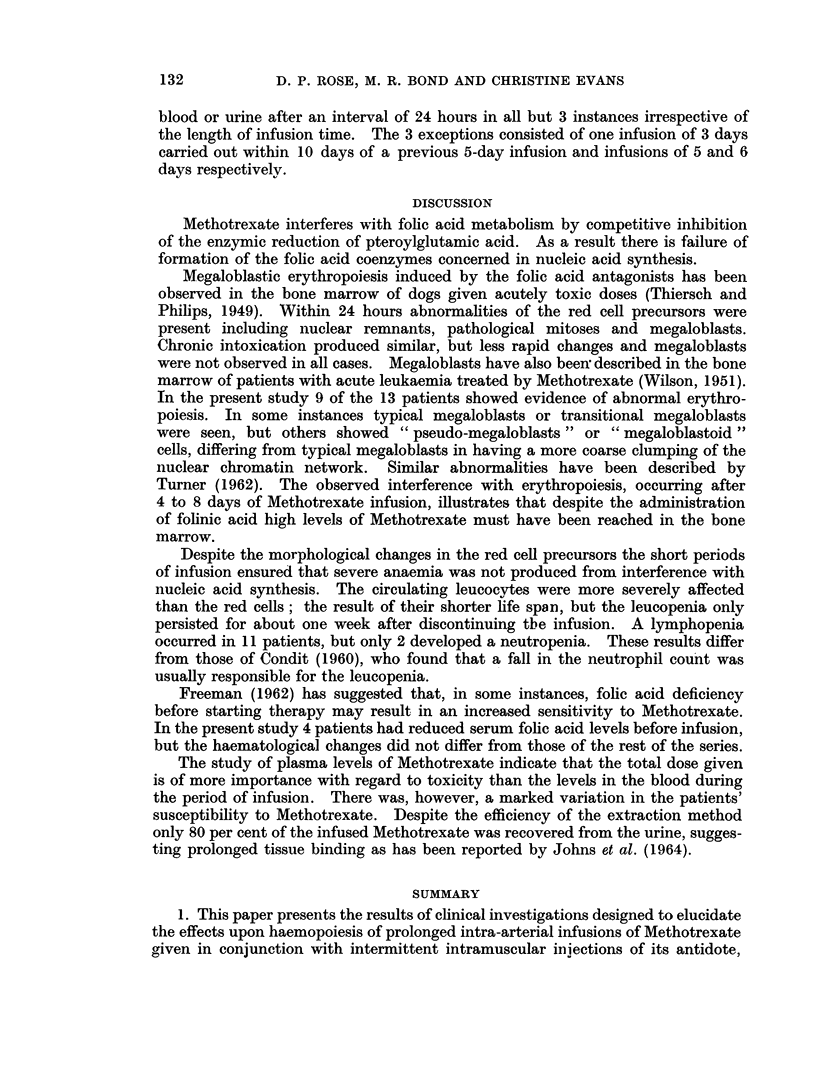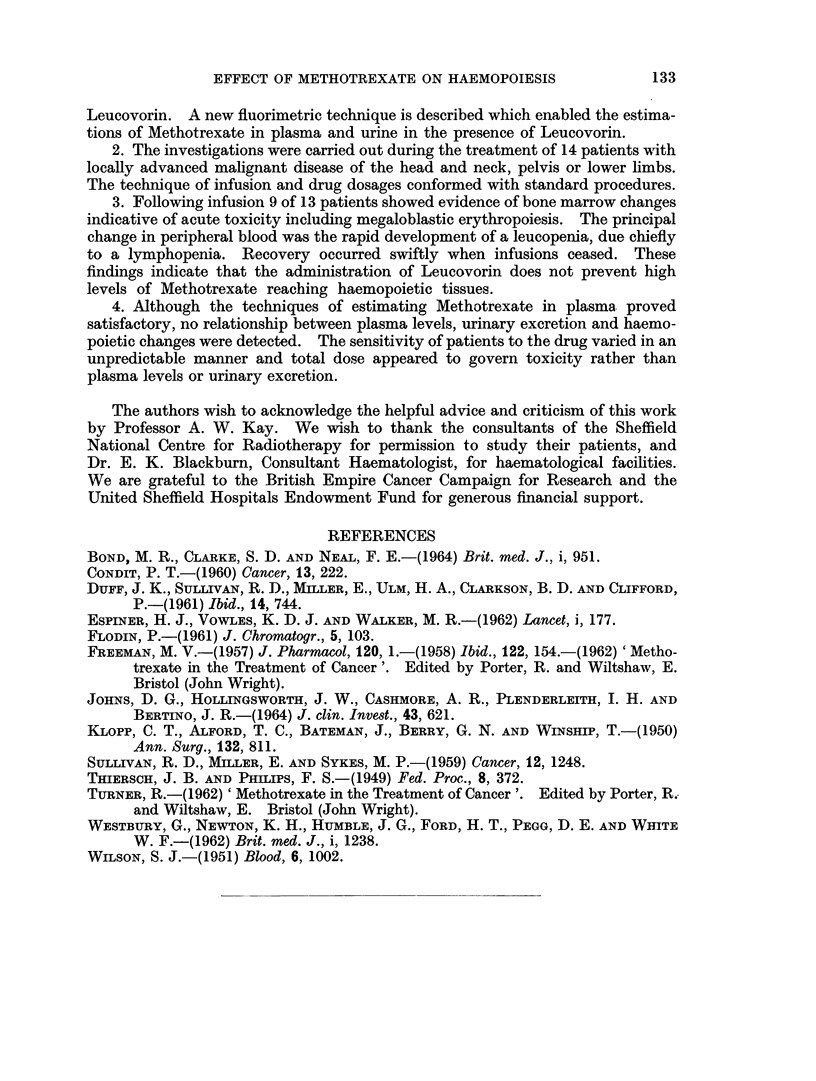# The Effects Upon Haemopoiesis of Prolonged Intra-arterial Infusions of Methotrexate Combined with the Intermittent Administration of Folinic Acid

**DOI:** 10.1038/bjc.1965.14

**Published:** 1965-03

**Authors:** D. P. Rose, M. R. Bond, Christine Evans


### THE EFFECTS UPON HAEMOPOIESIS OF PROLONGED INTRA-ARTERIAL INFUSIONS OF METHOTREXATE COMBINED WITH THE INTERMITTENT ADMINISTRATION OF FOLINIC ACID

### WITH OBSERVATIONS ON THE PLASMA LEVELS AND URINARY EXCRETION OF METHOTREXATE

D. P. ROSE,* M. R. BOND† AND CHRISTINE EVANS

*From the University Department of Surgery and The Department of Haematology, The Royal Infirmary, Sheffield*

Received for publication November 16, 1964

FOLLOWING the description by Klopp and his colleagues (1950) of the treatment of locally advanced malignant disease by the intra-arterial injection of nitrogen mustard, considerable interest has been taken in intravascular techniques developed for the administration of cytotoxic agents. Sullivan, Miller and Sykes (1959) first described the administration of the folic acid antagonist Methotrexate (amethopterin, 4-amino-$N^{10}$ methyl pteroylglutamic acid) by continuous intra-arterial infusion combined with the intra-muscular administration of folinic acid (Leucovorin). Using this technique it became possible to infuse the tumour area with a high concentration of the drug for long periods, its severe toxic effects being prevented by the folinic acid. Variable degrees of therapeutic success have been reported (Duff *et al.*, 1961 ; Westbury *et al.*, 1962 ; Espiner, Vowles and Walker, 1962), but there is little detailed information on the effect of such infusions upon haemopoiesis.

Freeman (1958, 1962) has studied plasma and urine levels of Methotrexate following oral and intravascular administration, but the patients were not receiving folinic acid and the periods of treatment were short. The lack of a method for the estimation of Methotrexate in the presence of folinic acid has previously prevented a study of the plasma levels attained during prolonged infusion, using the combined therapy procedure, and their relationship to haematological changes, local toxicity and clinical results.

In the present study patients with locally advanced malignant disease have been treated by continuous intra-arterial infusion of Methotrexate combined with the intermittent, intramuscular administration of folinic acid. A fluorimetric technique for the estimation of plasma levels of Methotrexate developed by one of us (C. Evans) has enabled the plasma levels, together with the urinary excretion, to be related to the changes observed in the bone marrow and peripheral blood.

### MATERIAL AND METHODS

Fourteen patients with locally recurrent malignant disease in the head and neck region, the pelvis and the lower limbs were studied (Table I).

* Present address: University Department of Chemical Pathology, Sheffield
† In receipt of a grant from the British Empire Cancer Campaign for Research.

TABLE I.—*Composition of Patients*

| Case Number | Sex | Age | Diagnosis | Previous therapy | Duration of Infusion Days |
|---|---|---|---|---|---|
| 1 | M | 48 | Squamous cell carcinoma penis | Surgery Radiation | 7 |
| 2 | F | 44 | Squamous cell carcinoma cheek | Surgery Radiation | 8 |
| 3 | F | 44 | Squamous cell carcinoma cervix | Radiation | 6 |
| 4 | M | 63 | Myxosarcoma of thigh | Surgery | 4 |
| 5 | F | 57 | Adenocarcinoma of colon | Surgery Radiation | 4 |
| 6 | F | 40 | Squamous cell carcinoma cervix | Radiation | 5 |
| 7 | F | 67 | Squamous cell carcinoma tongue | Radiation | 5 |
| 8 | F | 54 | Squamous cell carcinoma tongue | Surgery Radiation | 5 |
| 9 | M | 47 | Mixed salivary gland tumour | Surgery Radiation | 5 and 3 |
| 10 | F | 64 | Adenocarcinoma of rectum | Nil | 6 |
| 11 | F | 56 | Squamous cell carcinoma anus | Nil | 5 |
| 12 | M | 56 | Squamous cell carcinoma scrotum | Surgery Radiation | 6 |
| 13 | M | 63 | Papillary carcinoma bladder | Radiation | 6 |
| 14 | M | 41 | Adenocarcinoma of rectum | Nil | 5 |

Arterial catheterisation was performed by a technique previously described (Bond, Clarke and Neal, 1964).

Methotrexate was administered in a total dose of 50 mg. daily by continuous infusion using a constant infusion pump (Distillers Co. Ltd. ; Micro Type " S "). Doses of 25 mg. in 480 ml. sterile distilled water were given in each 12-hour period. Folinic acid was given intramuscularly throughout the period of infusion and for the subsequent 48 hours in a dose of 6 mg. every 6 hours. Infusions varied in length from 3 to 8 days depending upon the severity of local and systemic toxicity. In 2 cases accidental withdrawal of the catheter shortened the proposed course of treatment.

Haemoglobin estimations and leucocyte and platelet counts were carried out daily during the period of infusion and then until the values had returned to normal. Bone marrow examinations were performed before infusion in 6 patients and within 48 hours after infusion in all but one.

In 10 patients the fasting serum folic acid activity (*Lactobacillus casei* method : normal range 5 to 22 m$\mu$g. per ml.) was determined before, and in 5 patients repeated 24 to 48 hours after infusion.

Using the method of Freeman (1958), the concentration of Methotrexate was estimated in 24-hour urine samples collected throughout infusion and for the 2 subsequent 24-hour periods. By a modification of the method of Freeman (1957), the concentration of Methotrexate in plasma was estimated at 24-hour intervals during and up to 48 hours after infusion.

### *Modified Method for Estimation of Methotrexate in Plasma*

*Principle*

Following the separation of Methotrexate from the plasma proteins the increase in fluorescence of Methotrexate on oxidation was measured. The fluorescence excitation spectra (uncorrected) of oxidised Methotrexate and oxidised folinic acid are shown in Fig. 1. At an excitation wavelength of 370 mμ both oxidised components have a similar fluorescence emission but at an excitation wavelength of 382 mμ oxidised Methotrexate has a fluorescence emission, but there is no detectable emission from oxidised folinic acid. The increase in the fluorescence of Methotrexate on oxidation can therefore be measured at excitation wavelength 382 mμ without interference due to folinic acid.

Fig. 1.—The fluorescence excitation spectra (uncorrected) of oxidised Methotrexate (A) and oxidised folinic acid (B).

*Method*

Plasma was obtained from oxalated blood and Methotrexate then separated from the plasma proteins by gel-filtration on Sephadex G-25 (Pharmacia, Uppsala, Sweden). 1·2 g. of dry Sephadex G-25 was prepared in a water-swollen particulate form and used as a column 7·5 cm. long and 1 cm. diameter, according to the method of Flodin (1961). The bed was stabilised over 1 to 2 hours with distilled water before use.

The elution volumes for plasma proteins and Methotrexate were determined by passing 1 ml. of normal plasma and 1 ml. of a standard Methotrexate solution (1 mg. per ml.) through the column, and eluting with distilled water. Fractions of 1 ml. were collected and the absorbency of proteins at 280 mμ and Methotrexate at 370 mμ determined (Fig. 2). Methotrexate was eluted in fractions 8–15, in a total volume of 7 ml. The elution diagrams showed that whilst the Methotrexate fractions (8–15) and the plasma protein fractions (3–7) were effectively separated, a compound in the plasma absorbing at 280 m$\mu$ was eluted with the Methotrexate, but did not interfere with the assay.

1 ml. of plasma was used for each estimation. After separation of the Methotrexate fraction on the Sephadex column 0·1 ml. of 5 M acetate buffer was added to the 7 ml. eluate and the fluorescence (A) measured. The buffered eluate was oxidised with 0·1 ml. of 4 % $KMnO_4$ and, after standing for 5 minutes, 0·1 ml. of 3 % $H_2O_2$ was added and the fluorescence of Methotrexate measured after 2 minutes

FIG. 2.—Elution diagram showing the absorbency of proteins at 280 m$\mu$ (A) and of Methotrexate at 370 m$\mu$ (B).

(B). The increase in fluorescence on oxidation was therefore (B — A). A plasma sample obtained before any Methotrexate had been given was used as a blank. The fluorescence of Methotrexate in the sample was therefore (B — A) sample — (B — A) blank. Standard reference curves of the variation of fluorescence intensity with the concentration of Methotrexate were determined by this method.

### RESULTS

### *Haemoglobin levels*

The haemoglobin levels before infusion and the lowest value recorded during the period of infusion and for the subsequent 7 days are given in Table II. Eight of the 14 patients showed a fall of haemoglobin level greater than 2 g. per 100 ml.,

TABLE II.—*Haematological Data*

| Case Number | Hb. level g./100 ml. Pre-infusion | Hb. level g./100 ml. Lowest level | Total WBC/cu.mm. Pre-infusion | Total WBC/cu.mm. Lowest count | Lowest lymph. count/cu.mm. | Platelet count per cu.mm. Pre-infusion | Platelet count per cu.mm. Lowest count | Erythropoiesis post-infusion | Serum folate levels pre-infusion mμg./ml. |
|---|---|---|---|---|---|---|---|---|---|
| 1 | 10·6 | 7·9 | 20,000 | 1,100 | 180 | 260,000 | 170,000 | Megaloblasts and pseudo-megaloblasts | 4·7 |
| 2 | 11·1 | 9·7 | 8,000 | 1,400 | 168 | 165,000 | 90,000 | Hypoplastic, normoblastic | 6·4 |
| 3 | 13·2 | 9·3 | 11,000 | 2,000 | 294 | 200,000 | 60,000 | Normoblastic | — |
| 4 | 13·5 | 11·4 | 5,000 | 2,800 | 1,456 | 255,000 | 120,000 | Hypoplastic, normoblastic | 18·8 |
| 5 | 10·7 | 10·4 | 6,000 | 3,000 | 900 | 265,000 | 130,000 | Transitional megalo-blasts and pseudo-megaloblasts | 3·0 |
| 6 | 10·4 | 10·0 | 6,000 | 3,000 | 1,320 | 275,000 | 165,000 | Pseudo-megaloblasts | 4·4 |
| 7 | 11·5 | 10·4 | 5,000 | 3,400 | 600 | 120,000 | 120,000 | Transitional megaloblasts | 5·0 |
| 8 | 14·1 | 11·5 | 10,000 | 3,400 | 680 | 230,000 | 180,000 | Transitional megalo-blasts and pseudo-megaloblasts | — |
| 9 | 15·1 | 12·4 | 7,000 | 3,500 | 420 | 225,000 | 195,000 | — | 4·1 |
| 10 | 13·1 | 9·9 | 17,000 | 4,000 | 600 | 225,000 | 150,000 | Transitional megaloblasts | 10·0 |
| 11 | 12·3 | 8·5 | 9,000 | 6,000 | 480 | 430,000 | 125,000 | Transitional megaloblasts | 8·0 |
| 12 | 15·8 | 13·6 | 11,000 | 6,000 | 660 | 160,000 | 160,000 | Pseudo-megaloblasts | — |
| 13 | 10·9 | 10·9 | 8,000 | 6,000 | 540 | 165,000 | 165,000 | Normoblastic | 7·4 |
| 14 | 13·5 | 12·6 | 13,000 | 8,000 | 2,700 | 460,000 | 130,000 | Hypocellular Megaloblastic | — |

and of these 5 became mildly anaemic. In 1 further case (No. 1 in Table I) severe anaemia developed (Hb 7·9 g. per 100 ml.). This patient, however, was mildly anaemic before infusion and the bone marrow showed toxic changes.

*Leucocyte counts*

The total leucocyte count for each patient and the lowest count observed during or after infusion are given in Table II. Nine patients developed leucopenia (total count less than 4000 per cu. mm ) within 3 days of the end of infusion, but a rise to normal values took place during the next 7 days. A lymphopenia (less than 1000 lymphocytes per cu. mm.) was observed in 11 patients, in 4 of whom the total leucocyte count remained within the normal range (Table II). A neutropenia (less than 1500 neutrophil leucocytes per cu. mm.) occurred in only 2 cases.

*Platelet counts*

Although definite falls in the platelet counts were observed in 6 cases, thrombocytopenia (less than 100,000 per cu. mm.) occurred in only 2 cases.

*Bone marrow examination*

All bone marrow examinations performed before infusion showed normoblastic erythropoiesis. Samples of bone marrow from 9 of 13 patients examined after infusion contained megaloblasts, pseudomegaloblasts, or transitional megaloblasts and giant metamyelocytes. Three showed solely normoblastic erythropoiesis, but in 2 of these the marrow was hypoplastic.

*Serum folic acid activity*

The results for 10 patients in whom the serum folic acid activity was assayed before infusion are included in Table II. Four patients had levels below the normal range, in two of whom bone marrow examination showed normoblastic erythropoiesis.

Estimations of serum folic acid activity were carried out within 48 hours of infusion in 5 patients and in each the serum failed to support the growth of *Lactobacillus casei*. Bone marrow examinations performed in 4 of these patients showed megaloblastic erythropoiesis.

*Methotrexate levels in plasma and urine*

The daily plasma concentrations and the 24-hour urinary excretions of Methotrexate are given in Table III. In general the plasma concentrations showed a rise

TABLE III.—*The Plasma and Urinary Concentrations of Methotrexate During and After Infusion*

| Patient | | Days 1 | 2 | 3 | 4 | 5 | 6 | 7 | 8 | 9 | 10 |
|---|---|---|---|---|---|---|---|---|---|---|---|
| 2 | P | — | 2·70 | 3·30 | 3·60 | 1·70 | 1·80 | 1·30 | 1·30 | *2·20* | *0* |
| | U | 26·60 | 88·50 | 71·50 | 0 | 0 | 88·0 | 44·0 | 23·4 | *0* | *0* |
| 3 | P | — | 1·65 | 0·85 | 0·90 | 0·50 | 1·30 | *1·25* | *0* | | |
| | U | 19·50 | 32·40 | 37·60 | 53·0 | 63·0 | 40·0 | *19·5* | *0* | | |
| 6 | P | 0·45 | 1·55 | — | 0·20 | 0·60 | *0·40* | *0* | | | |
| | U | 52·2 | 43·2 | 50·0 | 50·8 | 38·6 | *21·4* | *0* | | | |
| 7 | P | — | — | 1·20 | 0·40 | 0·80 | 1·30 | *0* | *0* | | |
| | U | 5·40 | 14·40 | 67·50 | 63·50 | 60·20 | *35·30* | *0* | *0* | | |
| 8 | P | — | — | 0·55 | 0 | 0·30 | *0·15* | *0* | *0* | | |
| | U | 4·90 | 5·20 | 29·20 | 16·0 | 70·30 | *52·30* | *19·60* | *0* | | |
| 9 | P | -- | 4·20 | 0·30 | 0·85 | 0·45 | *0·05* | *0* | | | |
| 9[1] | U | 19·2 | 28·0 | 47·3 | 102·0 | 13·9 | *0* | *0* | | | |
| 9[2] | P | 0·85 | 0·60 | 0 | *0·85* | *0·40* | *0* | *0* | | | |
| | U | 0 | 38·0 | 27·2 | *33·6* | *3·35* | *1·0* | *0* | | | |
| 10 | P | — | 4·20 | 2·10 | 1·00 | 1·40 | 1·50 | *1·10* | *0* | | |
| | U | 23·1 | 81·0 | 67·0 | 14·9 | 65·0 | 44·0 | — | *0* | | |
| 11 | P | — | 1·35 | 3·60 | 1·20 | *0* | *0* | | | | |
| | U | 61·0 | 38·0 | 50·0 | 28·8 | 27·4 | *0* | | | | |
| 12 | P | — | 1·0 | 1·0 | 0·75 | 0 | 1·35 | *0·8* | *0* | *0* | |
| | U | 0 | 53·0 | 32·0 | 45·0 | 44·0 | 97·0 | *100·0* | *53·0* | *0* | |
| 13 | P | — | 1·05 | 2·80 | 1·60 | 1·80 | 0·40 | *0·60* | | | |
| | U | 35·8 | — | 34·5 | 61·7 | 57·5 | *48·8* | — | | | |

*Note:* Figures in italics indicate levels after infusion.
P = Plasma concentration μg./ml.
U = Urinary excretion mg./24 hours.

until the second or third day of infusion and then remained at approximately the same level until infusion ceased. The rates of urinary excretion showed a similar pattern ; increasing over a period of 3 to 4 days and then either remaining steady or diminishing slightly. Following infusion, Methotrexate was not detected in blood or urine after an interval of 24 hours in all but 3 instances irrespective of the length of infusion time. The 3 exceptions consisted of one infusion of 3 days carried out within 10 days of a previous 5-day infusion and infusions of 5 and 6 days respectively.

### DISCUSSION

Methotrexate interferes with folic acid metabolism by competitive inhibition of the enzymic reduction of pteroylglutamic acid. As a result there is failure of formation of the folic acid coenzymes concerned in nucleic acid synthesis.

Megaloblastic erythropoiesis induced by the folic acid antagonists has been observed in the bone marrow of dogs given acutely toxic doses (Thiersch and Philips, 1949). Within 24 hours abnormalities of the red cell precursors were present including nuclear remnants, pathological mitoses and megaloblasts. Chronic intoxication produced similar, but less rapid changes and megaloblasts were not observed in all cases. Megaloblasts have also been described in the bone marrow of patients with acute leukaemia treated by Methotrexate (Wilson, 1951). In the present study 9 of the 13 patients showed evidence of abnormal erythropoiesis. In some instances typical megaloblasts or transitional megaloblasts were seen, but others showed " pseudo-megaloblasts " or " megaloblastoid " cells, differing from typical megaloblasts in having a more coarse clumping of the nuclear chromatin network. Similar abnormalities have been described by Turner (1962). The observed interference with erythropoiesis, occurring after 4 to 8 days of Methotrexate infusion, illustrates that despite the administration of folinic acid high levels of Methotrexate must have been reached in the bone marrow.

Despite the morphological changes in the red cell precursors the short periods of infusion ensured that severe anaemia was not produced from interference with nucleic acid synthesis. The circulating leucocytes were more severely affected than the red cells ; the result of their shorter life span, but the leucopenia only persisted for about one week after discontinuing the infusion. A lymphopenia occurred in 11 patients, but only 2 developed a neutropenia. These results differ from those of Condit (1960), who found that a fall in the neutrophil count was usually responsible for the leucopenia.

Freeman (1962) has suggested that, in some instances, folic acid deficiency before starting therapy may result in an increased sensitivity to Methotrexate. In the present study 4 patients had reduced serum folic acid levels before infusion, but the haematological changes did not differ from those of the rest of the series.

The study of plasma levels of Methotrexate indicate that the total dose given is of more importance with regard to toxicity than the levels in the blood during the period of infusion. There was, however, a marked variation in the patients' susceptibility to Methotrexate. Despite the efficiency of the extraction method only 80 per cent of the infused Methotrexate was recovered from the urine, suggesting prolonged tissue binding as has been reported by Johns *et al.* (1964).

### SUMMARY

1. This paper presents the results of clinical investigations designed to elucidate the effects upon haemopoiesis of prolonged intra-arterial infusions of Methotrexate given in conjunction with intermittent intramuscular injections of its antidote,

Leucovorin. A new fluorimetric technique is described which enabled the estimations of Methotrexate in plasma and urine in the presence of Leucovorin.

2. The investigations were carried out during the treatment of 14 patients with locally advanced malignant disease of the head and neck, pelvis or lower limbs. The technique of infusion and drug dosages conformed with standard procedures.

3. Following infusion 9 of 13 patients showed evidence of bone marrow changes indicative of acute toxicity including megaloblastic erythropoiesis. The principal change in peripheral blood was the rapid development of a leucopenia, due chiefly to a lymphopenia. Recovery occurred swiftly when infusions ceased. These findings indicate that the administration of Leucovorin does not prevent high levels of Methotrexate reaching haemopoietic tissues.

4. Although the techniques of estimating Methotrexate in plasma proved satisfactory, no relationship between plasma levels, urinary excretion and haemopoietic changes were detected. The sensitivity of patients to the drug varied in an unpredictable manner and total dose appeared to govern toxicity rather than plasma levels or urinary excretion.

The authors wish to acknowledge the helpful advice and criticism of this work by Professor A. W. Kay. We wish to thank the consultants of the Sheffield National Centre for Radiotherapy for permission to study their patients, and Dr. E. K. Blackburn, Consultant Haematologist, for haematological facilities. We are grateful to the British Empire Cancer Campaign for Research and the United Sheffield Hospitals Endowment Fund for generous financial support.

### REFERENCES

Bond, M. R., Clarke, S. D. and Neal, F. E.—(1964) *Brit. med. J.*, i, 951.
Condit, P. T.—(1960) *Cancer*, **13**, 222.
Duff, J. K., Sullivan, R. D., Miller, E., Ulm, H. A., Clarkson, B. D. and Clifford, P.—(1961) *Ibid.*, **14**, 744.
Espiner, H. J., Vowles, K. D. J. and Walker, M. R.—(1962) *Lancet*, i, 177.
Flodin, P.—(1961) *J. Chromatogr.*, **5**, 103.
Freeman, M. V.—(1957) *J. Pharmacol*, **120**, 1.—(1958) *Ibid.*, **122**, 154.—(1962) ' Methotrexate in the Treatment of Cancer '. Edited by Porter, R. and Wiltshaw, E. Bristol (John Wright).
Johns, D. G., Hollingsworth, J. W., Cashmore, A. R., Plenderleith, I. H. and Bertino, J. R.—(1964) *J. clin. Invest.*, **43**, 621.
Klopp, C. T., Alford, T. C., Bateman, J., Berry, G. N. and Winship, T.—(1950) *Ann. Surg.*, **132**, 811.
Sullivan, R. D., Miller, E. and Sykes, M. P.—(1959) *Cancer*, **12**, 1248.
Thiersch, J. B. and Philips, F. S.—(1949) *Fed. Proc.*, **8**, 372.
Turner, R.—(1962) ' Methotrexate in the Treatment of Cancer '. Edited by Porter, R. and Wiltshaw, E. Bristol (John Wright).
Westbury, G., Newton, K. H., Humble, J. G., Ford, H. T., Pegg, D. E. and White W. F.—(1962) *Brit. med. J.*, i, 1238.
Wilson, S. J.—(1951) *Blood*, **6**, 1002.